# Delayed Presentation of Submucosal Retained Toothbrush from Self-Inflicted Injury in Patient with Schizophrenia

**DOI:** 10.1155/2017/2480140

**Published:** 2017-12-31

**Authors:** Caleb H. Creswell, Tony L. Kille, Matthew R. Hoffman, Tabassum Kennedy, Seth H. Dailey

**Affiliations:** ^1^Division of Otolaryngology-Head and Neck Surgery, Department of Surgery, University of Wisconsin School of Medicine and Public Health, Madison, WI, USA; ^2^Department of Radiology, Division of Neuroradiology, University of Wisconsin School of Medicine and Public Health, Madison, WI, USA

## Abstract

Foreign body ingestion occurs in not only children but also adults, particularly those with history of neurologic disease, alcohol use, or psychiatric disease. We present the case of a 40-year-old male with schizophrenia who presented to the emergency room with a long history of pharyngeal foreign body sensation which had recently progressed to include trismus, odynophagia, and dyspnea. Flexible laryngoscopy demonstrated fullness of the right posterior pharyngeal wall and computed tomography (CT) showed a linear opaque foreign body extending from the level of the oropharynx to the thyroid ala. Further history elicited that he stabbed himself in the pharynx two years prior with a toothbrush following a command hallucination. The toothbrush was removed uneventfully via an external approach. The patient was discharged with psychiatry follow-up. This case is unusual due to the submucosal location of the foreign body and the length of retention. It demonstrates the atypical nature which patients with comorbid psychiatric illness may present following foreign body injury and the use of an external surgical approach for the removal of a retained foreign body based on CT reconstruction.

## 1. Introduction

Following foreign body ingestion, 60% of patients will develop symptoms within 24 hours and 80% will do so within one week [1]. Interestingly, there is a small subset of patients in whom the foreign body can migrate over time, leading to delayed and much more subtle presentation [2–4]. In those patients, sensation of a foreign body or lump in the throat may be the primary symptom [5, 6]. Other potential etiologies for this symptom include gastroesophageal reflux disease, postnasal drip, cricopharyngeal spasm, lingual tonsil hypertrophy, cervical osteophytes, and malignancy [5, 6]. Thorough history and flexible laryngoscopy are key components of the initial assessment.

Most cases of foreign body ingestion in children are accidental, while ingestion in adults may be related to neurologic dysfunction, trauma, alcohol use, or presence of a psychiatric disorder [7]. Detection of foreign body ingestion in adults with comorbid psychiatric illness may be challenging, particularly if delayed, and requires a high index of suspicion.

We present a case of foreign body ingestion in a patient with schizophrenia who had a long history of pharyngeal foreign body sensation who then developed more acute symptoms, prompting presentation to the emergency department two years after the initial incident.

## 2. Case Presentation

A 40-year-old male with a 20-year history of schizophrenia presented to the emergency department with long history of pharyngeal foreign body sensation which had progressed over one month to include sore throat, odynophagia, and dyspnea. Physical exam revealed asymmetric prominence of the right posterior oropharyngeal wall and trismus with maximal interincisural distance of two centimeters. There were tenderness, edema, and erythema of the right submandibular triangle without palpable mass.

**Figure 1:**
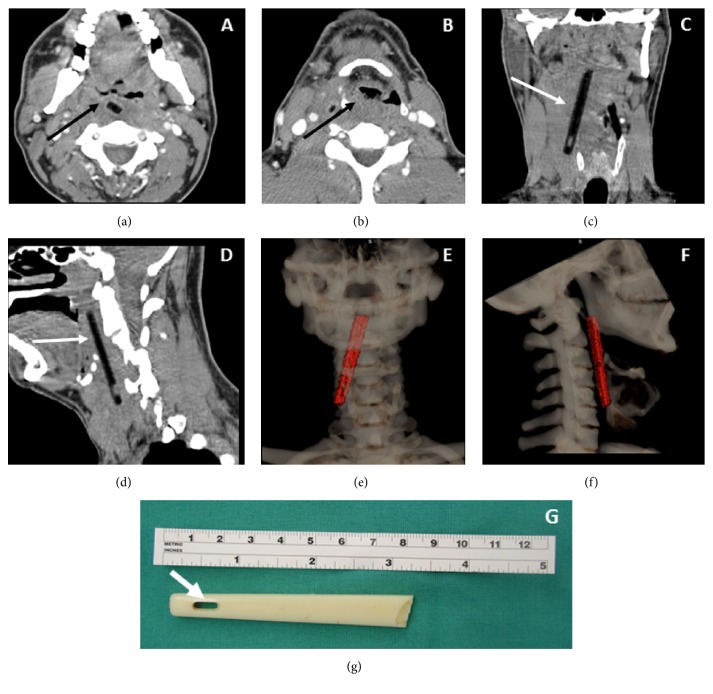
Two-dimensional axial (a, b), coronal (c), and sagittal (d) CT images and 3-dimensional coronal (e) and sagittal (f) reformats show a low density linear structure on the 2-dimensional images (white arrow) which is outlined in red on the 3-dimensional images. It is imbedded within the retropharyngeal soft tissues and extends from the level of the oropharynx through the hypopharynx to the right lateral extrapharyngeal soft tissues (black arrow). There is a linear gap at the end of the foreign body which represents the opening within the toothbrush handle (arrow (g)).

Flexible laryngoscopy showed fullness of the right posterior pharyngeal wall without mucosal abnormality, resulting in anterior displacement of the right aryepiglottic fold. This was encroaching on the airway but not causing critical airway stenosis. A computed tomography (CT) scan of the neck with contrast was obtained. This demonstrated a linear opacity extending from the oropharynx to the right thyroid ala. Reconstructed 3D images showed a uniform mass consistent with a foreign body (Figure 1). Further psychiatric history was obtained and revealed a history of auditory hallucinations that had on several occasions led to suicide attempts, including one attempt two years ago in which the patient stabbed himself in the pharynx with a broken toothbrush handle. Prompt removal was recommended given risk for infection and airway compromise.

**Figure 2:**
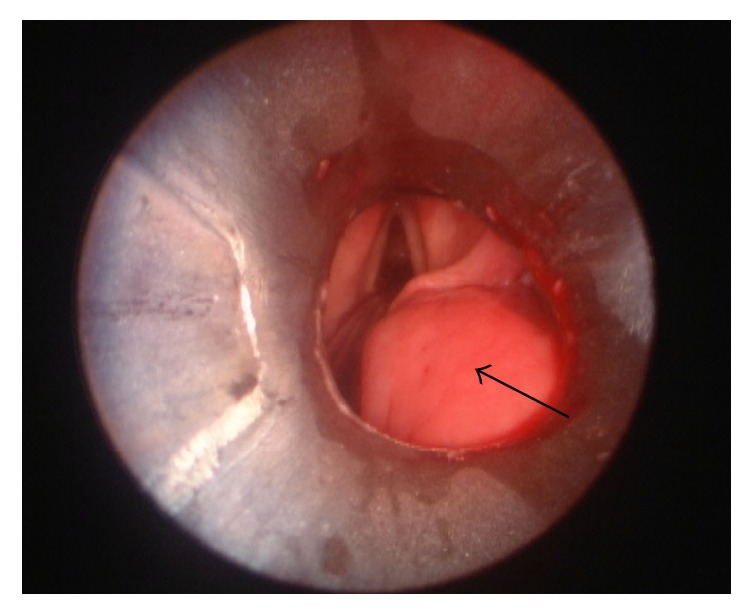
Photograph obtained during direct laryngoscopy showing fullness of the posterior right oropharynx and hypopharynx (arrow).

**Figure 3:**
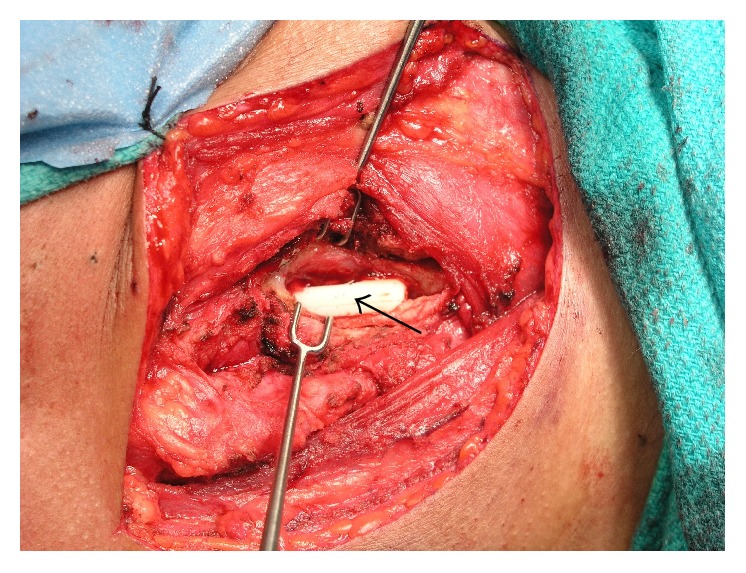
After raising subplatysmal flaps and entering a fibrous capsule, the toothbrush handle was visualized (arrow).

The patient was transorally intubated over a flexible bronchoscope and then underwent direct laryngoscopy. Swelling of the right posterior oropharynx and hypopharynx was noted (Figure 2). An anterolateral neck incision was made and subplatysmal flaps were raised. The mass was easily palpated overlying the right thyroid ala, and blunt dissection allowed entry into the fibrous capsule surrounding the foreign body (Figure 3). The capsule was opened, and the foreign body was removed. The length corresponded exactly to the length of the foreign body on the CT (Figure 1). A sample was obtained for bacterial culture, the wound was irrigated, and a drain was placed. The patient was extubated and given a soft diet.

Visual inspection and pathologic analysis confirmed that the foreign body was indeed a toothbrush handle. Cultures from the area grew heavy mixed bacteria including anaerobes. After an uneventful postoperative stay, the patient was discharged without complications. Discharge planning included psychiatry follow-up.

## 3. Discussion

Foreign body injuries are common, but the submucosal location, surgical planning for removal, and suspicion required to determine that a foreign body was present make this an interesting case. There is only one other similar report; a 16-month-old girl who had a toothbrush extracted from her posterior hypopharynx two months after a fall [8].

The submucosal location of the retained foreign body in this case is unusual. Foreign bodies can migrate submucosally when ischemic or traumatic damage leads to inflammation and granulation tissue that envelops the foreign body and incorporates it into the submucosa. Continuing pressure and ongoing granulation response allow for further migration [9]. In our case, the stabbing provided the initial traumatic damage which was then likely followed by inflammation and granulation response.

Surgical removal of an extraluminal foreign body requires precise anatomic information best provided by CT [10]. In this case, the 3D CT reconstruction motivated us to pursue an open approach for removal. Endoscopic removal would have required violation of an otherwise intact posterior pharyngeal wall and increased the risk of hypopharyngeal edema and airway obstruction. Measurement of foreign body length on CT also allowed us to confirm the entire foreign body had been removed. The majority of extraluminal aerodigestive foreign bodies are removed by an external surgical approach [10], though superficial foreign bodies can sometimes be removed endoscopically [11].

Important to this case was the increased index of suspicion required to determine that a foreign body was causing the patient's symptoms. Psychiatric illness is a risk factor, particularly in the setting of psychosis with command hallucinations [12, 13]. Life-threatening conditions may present atypically in these patients [12]. For our patient, a targeted history combined with standard diagnostic tests including flexible laryngoscopy and CT led to safe removal via an external approach.
